# PVP/CS/*Phyllanthus emblica* Nanofiber Membranes for Dry Facial Masks: Manufacturing Process and Evaluations

**DOI:** 10.3390/polym14214470

**Published:** 2022-10-22

**Authors:** Jian-Hong Lin, Bing-Chiuan Shiu, Po-Wen Hsu, Ching-Wen Lou, Jia-Horng Lin

**Affiliations:** 1 Fujian Key Laboratory of Novel Functional Fibers and Materials, Minjiang University, Fuzhou 350108, China; 2 Laboratory of Fiber Application and Manufacturing, Department of Fiber and Composite Materials, Feng Chia University, Taichung 40724, Taiwan; 3 College of Material and Chemical Engineering, Minjiang University, Fuzhou 350108, China; 4 Department of Medical Research, China Medical University Hospital, China Medical University, Taichung 40402, Taiwan; 5 Advanced Medical Care and Protection Technology Research Center, College of Textile and Clothing, Qingdao University, Qingdao 266071, China; 6 Innovation Platform of Intelligent and Energy-Saving Textiles, School of Textile Science and Engineering, Tiangong University, Tianjin 300387, China; 7 Department of Bioinformatics and Medical Engineering, Asia University, Taichung 413305, Taiwan; 8 School of Chinese Medicine, China Medical University, Taichung 40402, Taiwan

**Keywords:** electrospinning, *Phyllanthus emblica* (*P. emblica*), dry facial masks, Polyvinylpyrrolidone (PVP), Chitosan (CS)

## Abstract

In the wake of increasing demands on skin health, we propose simple, natural, and safe dry facial masks that restrict melanin synthesis. *Phyllanthus emblica* (*P. emblica*) is made into powders via a low-temperature extraction and freeze-drying process to serve as a natural agent. Next, it is added to mixtures containing Polyvinylpyrrolidone (PVP) and Chitosan (CS), after which the blends are electrospun into PVP/CS/*P. emblica* nanofiber membrane dry facial masks using the electrospinning technique. The dry facial masks are evaluated using the calibration analysis method, extraction rate test, scanning electron microscopy (SEM), release rate test, tyrosinase inhibition assay, biocompatibility test, and anti-inflammatory capacity test. Test results indicate that when the electrospinning mixture contains 29.0% *P. emblica*, the nanofibers have a diameter of ≤214.27 ± 74.51 nm and a water contact angle of 77.25 ± 2.21. *P. emblica* is completely released in twenty minutes, and the tyrosinase inhibition rate reaches 99.53 ± 0.45% and the cell activity ≥82.60 ± 1.30%. Moreover, the anti-inflammatory capacity test results suggest that dry facial masks confine inflammatory factors. PVP/CS/*P. emblica* nanofiber dry facial masks demonstrate excellent tyrosinase inhibition and are hydrophilic, biocompatible, and inflammation-free. The dry facial masks are a suitable material that is worthwhile exploring and applying to the cosmetic field.

## 1. Introduction

Nowadays, the majority of facial masks are commonly made of cellulous or polymer matrices (i.e., carriers) with a liquid blend that consists of an active substance. To achieve the purposes of forming, sale, and preservation, the effective substance is usually composed of chemical or synthetic elements. Meanwhile, the liquid in facial masks is also incorporated with essence, moisturizer, thickener, and antiseptic agents, and these additives may harm the health and living environment of people [1,2,3,4,5]. However, the manufacturing process can be adjusted to improve this disadvantage. For example, the electrospinning technique is introduced to the cosmetic field in order to produce dry fabrics that contain no extra additives while retaining biocompatible and eco-friendly attributes. In addition, nanofiber membranes are used as the carrier in which effective substances can be directly applied, thereby attaining applications that are simple, convenient, eco-friendly, and safe. In particular, electrospinning has been well developed and it can produce continuous and resilient nanofiber membranes [6,7]. With variations in the components, electrospinning parameters, and subsequent treatments, nanofiber membranes present many advantages, e.g., a high porosity, a large specific surface area, and an even pore size distribution [8,9,10]. The current use of electrospinning nanofiber membranes includes separation and purification technology [11,12,13], tissue engineering [14,15,16], biomedical science [17,18,19], wound healing [20,21,22], and functional textiles [23,24]. To sum up, the employment of electrospinning embodies structural benefits and electrospinning nanofiber membranes are widely used as a carrier that bears functional substances. These favorable attributes make electrospinning nanofiber membranes an excellent material for facial masks.

Diverse studies on the protection, improvement, and repair of skins have been conducted due to a rise in the concerns regarding skin health. The exposure to sunshine or UV radiation in daily environments may inflict skin with unevenly presented solar lentigines, melanoma, or skin cancer. Usually, UV block products are helpful to prevent damage rendered by the sun. Another effective measure is to block the melanin synthesis, which also reduces the risk of dermal illness. The mechanism of confining tyrosinase is most commonly applied and azelaic acid, kojic acid, aloin, or arbutin is thus used for the growth of melanocytes to subside [25,26,27,28,29,30,31]. In this study, *P. emblica* is used to restrict the growth of tyrosinase in order to prevent melanin from growing. *P. emblica* is a tropical fruit that is pervasively planted in the countries of Southeast Asia, such as China, India, Vietnam, and Thailand, where people have had the edible fruit over thousands of years. With considerable nutritional value and an abundance of tannins (33% of its dry weight), vitamins (especially vitamin C), amino acids, minerals, phenolic acid, and flavonoids, the fruit are fatigue-, oxidation-, and inflammation-resistant, and can prevent tumors and diabetes [32,33,34,35,36,37,38]. Because of the presence of vitamin C and excellent oxidation resistance, *P. emblica* shows potential for melanin-confining and oxidation-resistant cosmetics. 

Polyvinylpyrrolidone (PVP) is a synthetic polymer consisting of 1-vinyl-2-pyrrolidone monomer that exhibits excellent solubility. PVP also interacts with multiple hydrophilic and hydrophobic materials, the features of which makes PVP suitable for the use of blending materials [39]. Chitosan (CS) is a modified polymer that is derived from the deacetylation process of chitin that exhibits excellent biocompatibility and mechanical stability. Moreover, chitosan and its derivatives also demonstrate antibacterial and antifungal activity [40,41]. Owing to biodegradability and low toxicity, both PVP and CS are applied to the cosmetics, food, and biomedicine fields [42,43,44]. In this study, *P. emblica* undergoes a low-temperature extraction and freeze-drying process to form powder. *P. emblica* powders serve as a natural agent and are added to PVP/CS blends, after which the blend is made into PVP/CS/*P. emblica* melanin-confining nanofiber membranes (i.e., dry facial masks) via electrospinning. One only needs to apply the dry facial mask to the face and then spray water over. The mask will dissolve and take effect accordingly. In addition to an efficient and convenient application, the dry facial masks are also natural, eco-friendly and safe, thus enriching the diversity and prospective of the facial masks.

## 2. Materials and Methods

### 2.1. Materials

The *Phyllanthus emblica* (*P. emblica*) is purchased from Yungtai Orchard Co., Ltd., Taichung, Taiwan. Polyvinylpyrrolidone (SIGMA, Uni-Onward Corp., New Taipei City, Taiwan) has a molecular weight of 360,000. Water soluble chitosan (Charming & Beauty Co., Ltd., Taichung, Taiwan) has a deacetylation of 91.1% and a molecular weight of 100,000. Alcohol has a concentration of 95%. The NIH/3T3 cells are purchased from the Bioresource Collection and Research Center (BCRC), Hsinchu, Taiwan. The Bovine calf serum (10%) and antibiotic are purchased from Gibco, Quantum Biotechnology., Inc., Taichung, Taiwan. The human TNF-α ELISA kit and human IL-1α ELISA kit are purchased from (Invitrogen) Quantum Biotechnology., Inc., Taichung, Taiwan.

### 2.2. Methods

#### 2.2.1. Extraction of *P. emblica*

The alcohol and deionized water (7:3 *v/v*%) are formulated into a solvent. Ten grams of *P. emblica* is weighed and then completely mixed with 100 mL of solvent, forming the *P. emblica* mixture. A magnet mixer is used to extract the *P. emblica* mixture at a temperature of 40 °C and an extraction rate of 100 rpm for six, twelve, twenty-four, and forty-eight hours. The impurities are removed from the extract with a piece of 0.22 µm filter paper and the filtrate is placed in a refrigerator at −20 °C overnight until it is frozen. Finally, the 24 h freeze-drying is employed to create *P. emblica* powders.

#### 2.2.2. Preparation of Nanofiber Membranes

**Figure 1:**
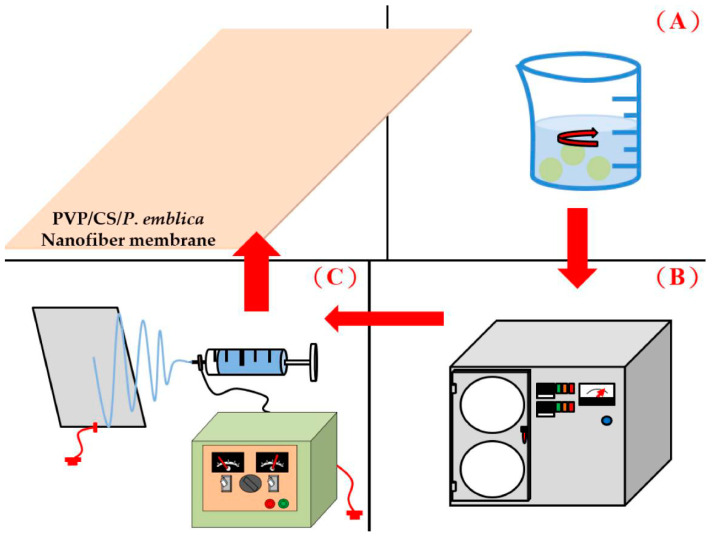
The manufacturing process of dry facial masks composed of nanofiber membranes: (**A**) the extraction process, (**B**) freeze-drying process, and (**C**) the preparation process of nanofiber membranes.

To begin, PVP (20 wt%) and CS (2 wt%) are separately processed at 60 °C for twelve hours using a magnet mixer. When both of them are dissolved completely, the PVP solution and CS solutions are blended for thirty minutes. Next, 0, 0.005, 0.01, and 0.02 wt% of the *P. emblica* powders are separately mixed with PVP/CS blends for thirty minutes using a magnet mixer, after which the ultrasonic vibration machine is used to remove the bubbles. Finally, the electrospinning process is conducted as follows. The PVP/CS/*P. emblica* mixtures are infused into a 15-mL syringe with needle #22. An electrospinning syringe pump (KD Scientific, KDS220, Sanit Louis, MO, USA) is equipped with a static generator (COSMI, SC-80H, Taichung, Taiwan). The mixture is pushed through the needle to form a Taylor cone and then drawn from the charged end by the electric field and drafted to the collect plate in another non-charged end. The repetitively collected electrospinning nanofibers are accumulated into nanofiber membranes. During the electrospinning process, the needle is designed to sway back and forth in order to broaden the collection range of the membranes. PVP/CS/*P. emblica* nanofiber membranes are thus formed and then stored in a moisture-proof case immediately. The spinning rate is 0.025 mL/min; the electrospinning distance is 15 cm; the electrospinning voltage is 15 kV; the temperature is 25–30 °C; and the humidity is 35%. Figure 1 shows the diagram of the process.

### 2.3. Characterizations 

#### 2.3.1. Extraction Rate Measurement 

The immersion method is used. *P. emblica* is weighed (W0) with a specified weight and then placed in a beaker for extraction. The extraction is conducted at a temperature of 40 °C with an extraction time of 6, 12, 24, and 48 h. The individual extraction solutions are freeze-dried into powders (Wt) and the extraction rate is computed with the equation as follows.

extraction rate (%) = (Wt/W0) × 100%.

#### 2.3.2. Scanning Electron Microscopy

Samples are affixed to the foundation of the scanning electron microscope (HITACHI S-4800, HORIBA EMAX400, Kyoto, Japan) with carbon tape, and observed for the surface morphology at an operation voltage of 15 kV.

#### 2.3.3. Water Contact Angle Test

Samples are measured with a water contact angle meter (OCA-15 PLUS, SCA20, Munich, Germany). Next, the test solution (i.e., deionized water) is used to fill a syringe, and the photograph device is adjusted to a suitable position. A sample is fixed on a plate beneath the syringe, after which 20 µL of deionized water is dripped over the sample surface. The photograph device is activated as soon as the drop contacts the nanofiber membrane. The highest point along with two points on both sides of the drop are measured and recorded for contact angle analysis. Six samples for each specification are used for the average. 

#### 2.3.4. Release Rate Measurement

Samples are trimmed into cubes of 1 cm × 1 cm, and then placed in a spectrometer tube filled with deionized water for specified lengths of time (e.g., 5, 10, 15, and 20 min). The samples are removed and measured for the optical density using a UV-Vis spectrophotometer (Thermo Fisher, Genesys 10S UV-Vis, Pleasanton, CA, USA). The calibration analysis linear regression is used to compute the release rate.

#### 2.3.5. Tyrosinase Inhibitor Assay

96-well plates are used to formulate solutions A, B, C, and D as in Table 1. The tyrosinase inhibition rate is computed with the equation as follows and vitamin C serves as the control group. 

Tyrosinase inhibition rate (%): (A − B) − (C − D)/(A − B) × 100% where A, B, C, and D separately means the optical density of corresponding solutions.

#### 2.3.6. Biocompatibility Measurement 

The NIH/3T3 cells are cultured in 96-well plates (5 × 103 cells/mL) for a 24 h culture in a carbon dioxide incubator. Next, the culture solution is removed and then replaced with the culture solutions in which samples were once immersed. The cells in the 96-well plates are once again cultured in a carbon dioxide incubator for twenty-four and seventy-two hours. Afterwards, the plates are removed and a PrestoBlue agent is added for a reaction in 15–20 min. With a specified optical density of 570, the Micro plate ELISA Reader (Tecan, Sunrise, Switzerland) is used for the measurement. The data are yielded and computed for cell viability as follows.

Cell Viability (%) = (Wt/A) × 100%

Wt refers to the optical density for different culture solutions while A refers to the optical density for the pure culture solution. 

#### 2.3.7. Anti-Inflammatory Capacity Measurement 

The IL-1α and TNF-α are separately used for the anti-inflammatory capacity. The NIH/3T3 cells are cultured in 96-well plates (5 × 103 cells/mL) for twenty-four hours in a carbon dioxide incubator. The culture solution is removed and then replaced with another culture solution in which the samples were once immersed, after which the cells are once again cultured in the carbon dioxide incubator for twenty-four and seventy-two hours. The cells in the 96-well plates are removed in order to remove 50 µL of supernatant fluid. The fluid is added to other 96-well plates, followed by 50 µL of antibody cocktail. Afterwards, the mixtures are oscillated at 400 rpm for one hour, and the solution is removed. After the plates are rinsed with wash buffer and 100 µL of TMB development solution is added, the plates are oscillated at 400 rpm in darkness for ten minutes. Finally, 100 µL of stop solution is added for interaction, and then a micro plate ELISA Reader (Tecan, Sunrise, Zurich, Switzerland) is used for the measurement with a specified OD of 450.

## 3. Results

### 3.1. Calibration Analysis of P. emblica Powders

**Figure 2:**
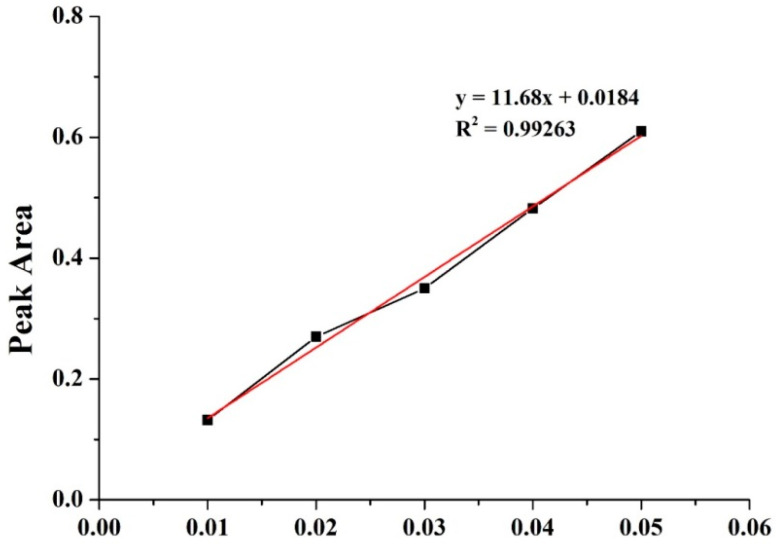
Calibration analysis of *P. emblica* powders.

Figure 2 shows the calibration analysis for powders of *P. emblica* extract as related to the concentrations of 0.01, 0.02, 0.03, 0.04, and 0.05 g. The samples are measured at a specified 490 nm using a UV-Vis spectrophotometer, and the corresponding optical density (OD) is 0.132, 0.27, 0.35, 0.482, and 0.61. According to the results, the OD is proportional to the *P. emblica* concentration, and R value (0.99263) also proves the accuracy of the test instrument.

### 3.2. Extraction Rate of P. emblica Powders

**Figure 3:**
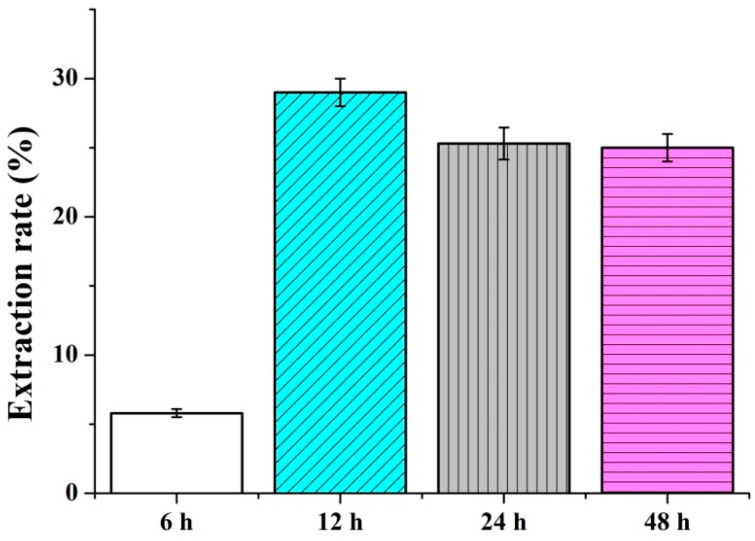
Extraction rate of *P. emblica* powders.

Figure 3 shows the solvent for the extraction of *P. emblica* is composed of water and alcohol. The weight of *P. emblica* is specified as ten grams and the extraction time is 6, 12, 24, or 48 h as in Table 2. The extract weight of powders is 0.58 g, 2.9 g, 2.53 g, and 2.5 g, respectively. The computation of extraction rate indicates that the corresponding extraction rates are 5.80 ± 0.30%, 29.00 ± 1.00%, 25.33 ± 1.15%, and 25.00 ± 1.00%.

### 3.3. Tyrosinase Inhibition Assay for P. emblica Powders

**Figure 4:**
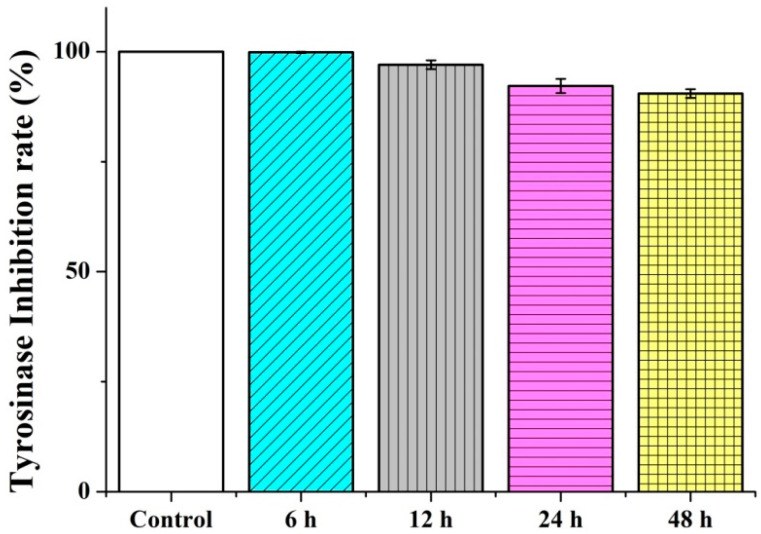
Tyrosinase inhibition rate of *P. emblica* as related to the extraction time.

As the presence of melanin is attributed with an interaction with tyrosinase, the restriction on tyrosinase is evaluated to examine whether the melanin synthesis is successfully blocked. *P. emblica* powders are evaluated for a tyrosinase inhibition rate. Tyrosinase is allowed to thaw in advance. Tyrosine, the extract, and tyrosinase are added to the 96-well plates in order, after which the tyrosinase inhibition rate is computed with the equation where the tyrosinase inhibition rate (%) is (A − B) − (C − D)/(A − B) × 100%. The tyrosinase inhibition rates are 99.83 ± 0.15%, 97.03 ± 0.97%, 92.20 ± 1.58%, and 90.46 ± 0.97% for the extraction times of six, twelve, twenty-four, and forty-eight hours, respectively. Vitamin C is used as the control group. Figure 4 shows that the Tyrosinase inhibition rate is dependent on the extraction time. There are more different extracts or impurities with an increase in the extraction time. With a specified concentration but a longer extraction time, the content of the tyrosinase inhibitor may be decreased, which in turn causes a decrease in the tyrosinase inhibition rate.

### 3.4. SEM Observation of Nanofiber Membranes

**Figure 5:**
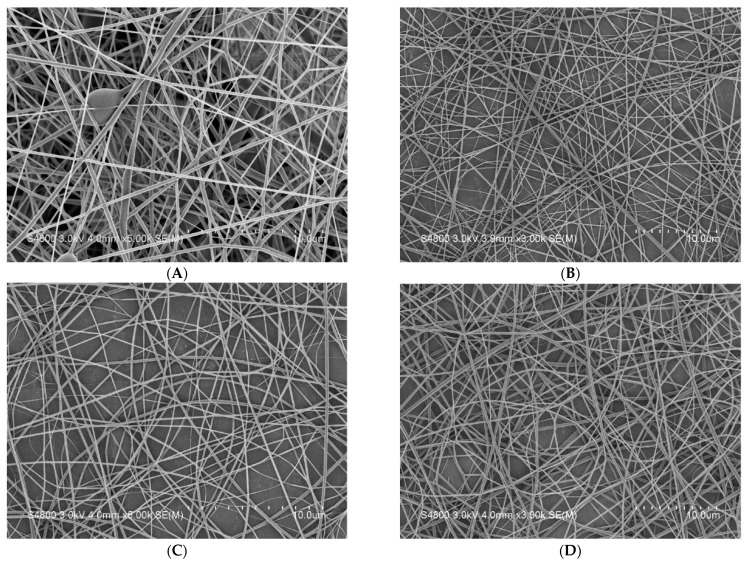

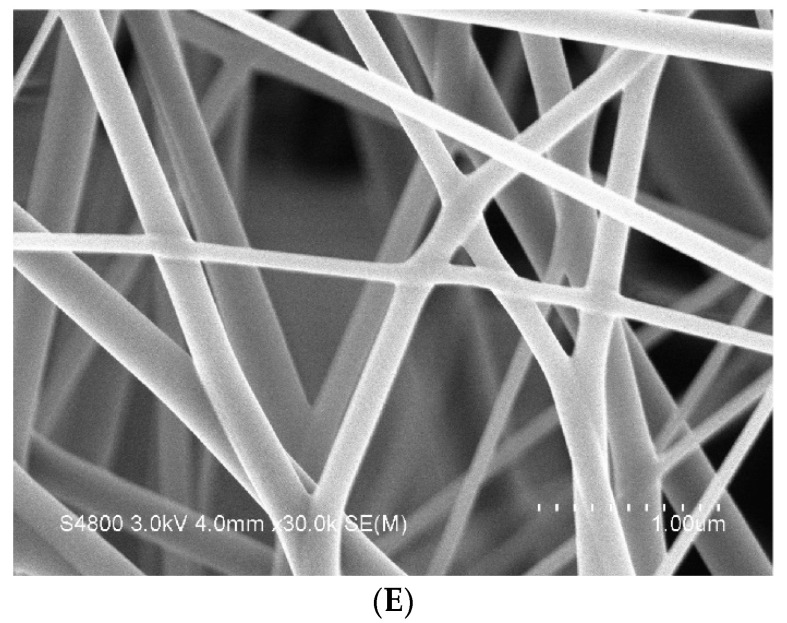
SEM images (magnification of 3 K) of PVP/CS/*P. emblica* nanofiber membranes of (**A**) PVP/CS/*P. emblica* 0 wt%, (**B**) PVP/CS/*P. emblica* 0.005 wt%, (**C**) PVP/CS/*P. emblica* 0.01 wt%, and (**D**) PVP/CS/*P. emblica* 0.02 wt%. (**E**) PVP/CS/*P. emblica* 0 wt% (magnification of 30 K).

The morphology, nanofiber diameter, and porosity of nanofiber membranes can be observed via the SEM images. Finer nanofibers possess good specific surface areas, thus enabling membranes to be well attached to skins as well facilitating the release of *P. emblica*. Meanwhile, a porous structure has a positive influence over the air ventilation. Based on Table 3 and Figure 5, PVP/WCS/*P. emblica* 0 wt%, PVP/WCS/*P. emblica* 0.005 wt%, PVP/WCS/*P. emblica* 0.01 wt%, and PVP/WCS/*P. emblica* 0.02 wt% individually have: fiber diameters of 214.27 ± 74.51 nm, 107.95 ± 27.74 nm, 90.32 ± 29.84 nm, and 165.33 ± 51.66 nm; viscosities of 351.8 cP, 397.1 cP, 421.6 cP, and 467.3 cP; and conductivities of 657 μS, 1084 μS, 1336 μS, and 1609 μS. The presence of *P. emblica* contributes toward a significant improvement in conductivity, which facilitates drafting during the electrospinning process, namely a significantly lower fiber diameter. In particular, the group of PVP/WCS/*P. emblica* 0.01 wt% exhibits the lowest nanofiber diameter. As for the group of PVP/WCS/*P. emblica* 0.02 wt%, the viscosity is increased, which subsequently changes the nanofiber diameter. Meanwhile, a rise in the nanofibers also means that there are *P. emblica* embedded. The variation in the nanofiber diameter is superior to that of PVP/WCS/*P. emblica* 0 wt% nanofiber membranes. Figure 5E presents a greater distinguish rate.

### 3.5. Hydrophilicity of Nanofiber Membranes 

**Figure 6:**
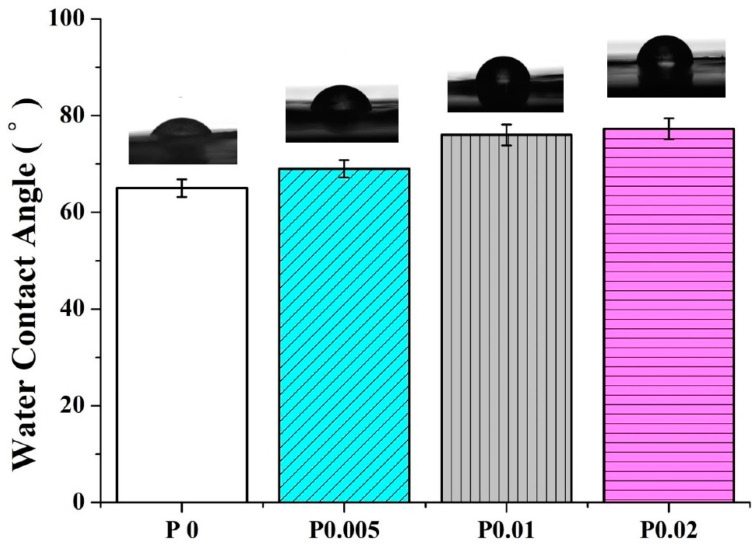
Hydrophilicity of nanofiber membranes as related to the *P. emblica* content.

The hydrophilicity of nanofiber membranes can be well perceived according to the water contact angle. Namely, a water contact angle lower than 90 degrees means the materials are hydrophilic and the lower the angle, the higher the hydrophilicity. In this study, the dry facial masks are different from the wet types that contain liquor components. To provide the dry facial masks with convenient and efficient uses, the masks require a certain hydrophilicity for purposes including moisture absorption, adhesion to skins, decomposition of masks, and the release of effective substances. Hence, the hydrophilicity for dry facial masks also affects the *P. emblica* release. Figure 6 shows that the water contact angles of PVP/WCS/*P. emblica* 0, PVP/WCS/*P. emblica* 0.005, PVP/WCS/*P. emblica* 0.01, and PVP/WCS/*P. emblica* 0.02 wt% are 65.00 ± 1.82°, 69.00 ± 1.82°, 76.00 ± 2.16°, and 77.25 ± 2.21°, respectively. PVP/WCS is a hydrophilic material and with a rise in the *P. emblica* content, the water contact angle of the membranes is also increased. When the water contact angle is lower than 90 degrees, the PVP/WCS/*P. emblica* 0 wt% nanofiber membranes still exhibit excellent hydrophilic properties.

### 3.6. Release Rate of Nanofiber Membranes

**Figure 7:**
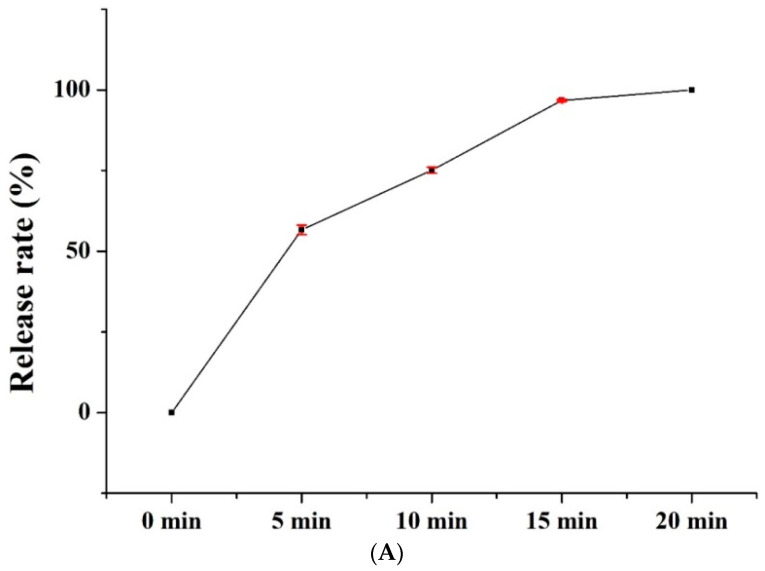

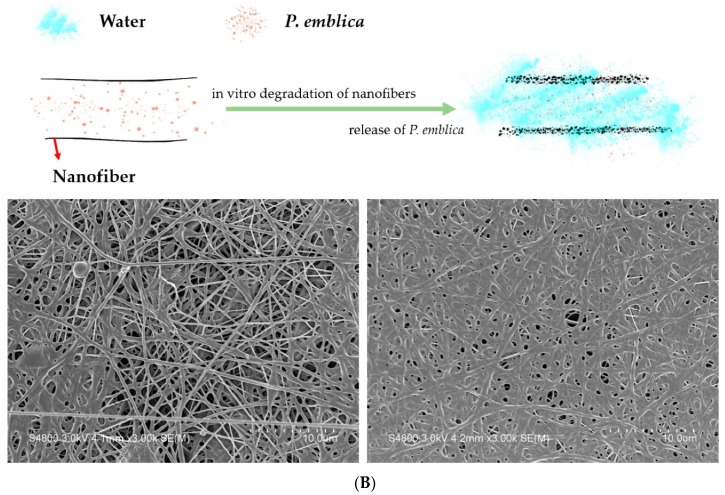
(**A**) Release rate of nanofiber membranes as related to the time and (**B**) SEM images of the releasing process and degrading process.

The PVP/WCS/*P. emblica* nanofiber membranes are tested for their release rates, which are 0 for PVP/WCS/*P. emblica* 0 wt%, 0.057 for PVP/WCS/*P. emblica* 0.005 wt%, 0.13 for PVP/WCS/*P. emblica* 0.01 wt%, and 0.27 for PVP/WCS/*P. emblica* 0.02 wt%. To sum up, the release rate is in direction proportion to the *P. emblica* content. Figure 7B demonstrates the release rate of PVP/WCS/*P. emblica* 0.02 wt% as related to different lengths of time. The release rates with corresponding times are 0% (0 min), 56.63 ± 1.49% (5 min), 75.10 ± 0.91% (10 min), 96.73 ± 0.35% (15 min), and 100.0 ± 0% (20 min). *P. emblica* is efficiently released in the first five minutes, then isometrically released in the subsequent 5–15 min, and eventually completely released in the final 15–20 min, thus demonstrating an excellent release rate curve as is required for facial masks. Figure 7B shows the diagram of releasing process of *P. emblica* from the nanofiber membranes. PVP/WCS/*P. emblica* nanofibers are decomposed in water, during which *P. emblica* is released from the nanofibers. The SEM image indicates the morphology when the nanofibers are decomposed. Nanofibers are gradually melted into membrane and then completely dissolved in water, due to which *P. emblica* is totally released in water.

### 3.7. Tyrosinase Inhibition Assay of Nanofiber Membranes

**Figure 8:**
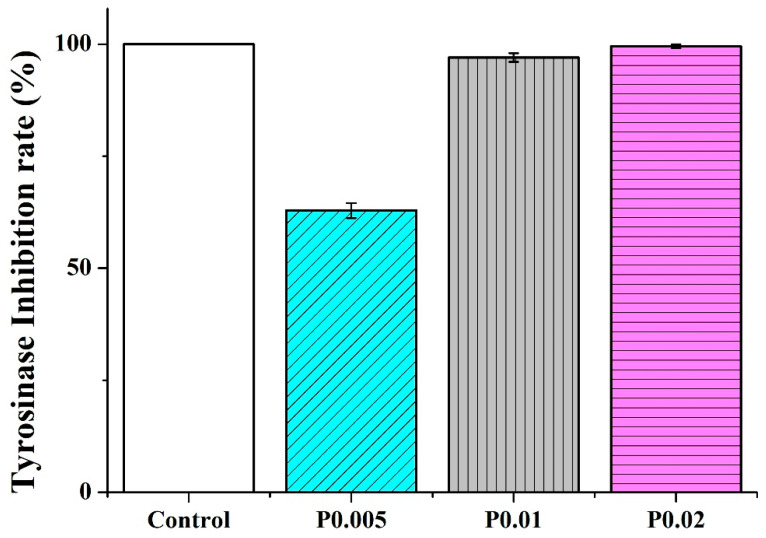
Tyrosinase inhibition rate of nanofiber membranes as related to the *P. emblica* content.

PVP/WCS/*P. emblica* nanofiber membranes are tested for their tyrosinase inhibition rate. Figure 8 shows that the control group (vitamin C) has an inhibition rate of 100% while the inhibition rates are 62.86 ± 1.68% for PVP/WCS/*P. emblica* 0.005 wt%, 97.03 ± 0.97% for PVP/WCS/*P. emblica* 0.01 wt%, and 99.53 ± 0.45% for PVP/WCS/*P. emblica* 0.02 wt%. To sum up, the tyrosinase inhibition rate is in direct proportion to the *P. emblica* content and reaches the optimal rate when the *P. emblica* content is 0.02 wt%. The strengthened activity of tyrosinase facilitates the transformation of tyrosine into levodopa (i.e., hydroxylation) and the series of reactions trigger the melanin synthesis. Consisting of tannins and vitamin C, *P. emblica* inhibits enzyme activity and then confines the melanin synthesis, which substantiates the incorporation of *P. emblica* with PVP/WCS nanofiber membranes that have a positive influence on confining the melanin synthesis [45].

### 3.8. Biocompatibility of Nanofiber Membranes

**Figure 9:**
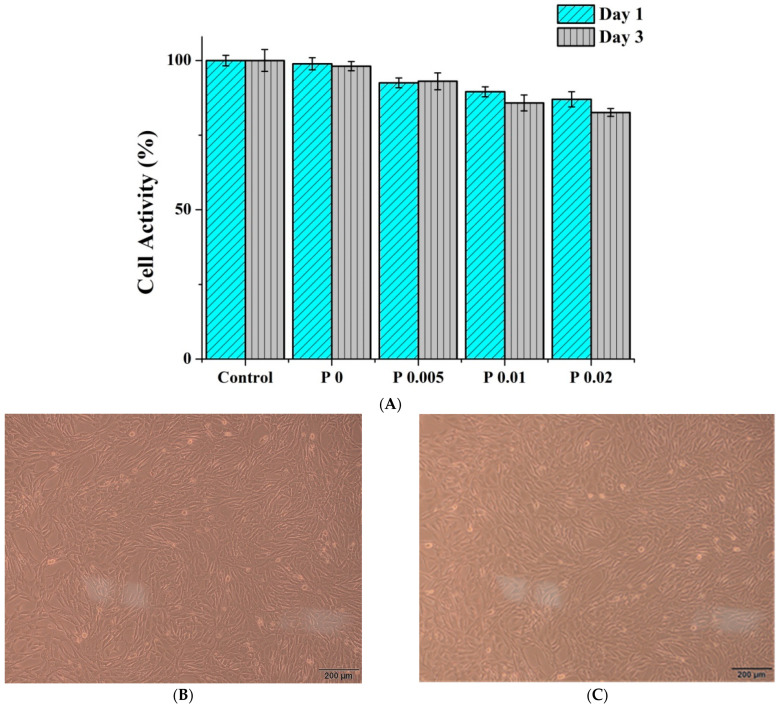

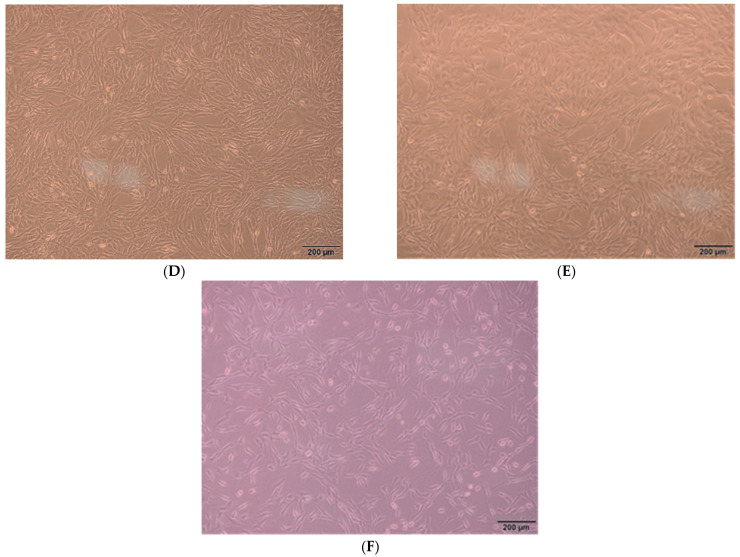
Biocompatibility of nanofiber membranes as related to the *P. emblica* content: (**A**) MTT cell activity, (**B**) PVP/CS/*P. emblica* 0 wt%, (**C**) PVP/CS/*P. emblica* 0.005 wt%, (**D**) PVP/CS/*P. emblica* 0.01 wt%, (**E**) PVP/CS/*P. emblica* 0.02 wt%, and (**B**–**F**) cytocompatibility test on day 3.

PVP/WCS/*P. emblica* nanofiber membranes are tested for cell viability. Figure 9 shows that the cell viability for the control group is 100%. On day 1, the PVP/WCS/*P. emblica* 0 wt%, PVP/WCS/*P. emblica* 0.005 wt%, PVP/WCS/*P. emblica* 0.01 wt%, and PVP/WCS/*P. emblica* 0.02 wt% showed cell viabilities of 98.92 ± 2.04%, 92.54 ± 1.64%, 89.53 ± 1.68, and 87.01 ± 2.56%, respectively. On day 3, the control group showed a cell viability of 100%, and the four groups show cell viabilities of 98.13 ± 1.54%, 93.06 ± 2.83%, 85.80 ± 2.65%, and 82.60 ± 1.30%. The higher the *P. emblica* content, the lower the cell viability. In sum, regardless of whether it is day 1 or day 3, the PVP/CS/*P. emblica* nanofiber membranes exhibit an average cell viability of over 80%, suggesting that the nanofiber membranes are biocompatible for cell growth [45]. The cytocompatiblity results in Figure 9B–F show the status of NIH/3T3 cells on day 3, the findings of which correspond to the cell activity. The PVP/WCS/*P. emblica* 0 wt% group shows that the dense cells are at a form of fine fusiform and firmly attached to the well. A small proportion of fibroblast cells have a round shape mainly because NIH/3T3 presents contact inhibition and an excessive number of cells trigger the contact inhibition. The cell density is inversely proportional to the content of *P. emblica* but still preserves the complete cell morphology and status, which confirms the cytocompatibility.

### 3.9. Anti-Inflammatory Capacity of Nanofiber Membranes 

**Figure 10:**
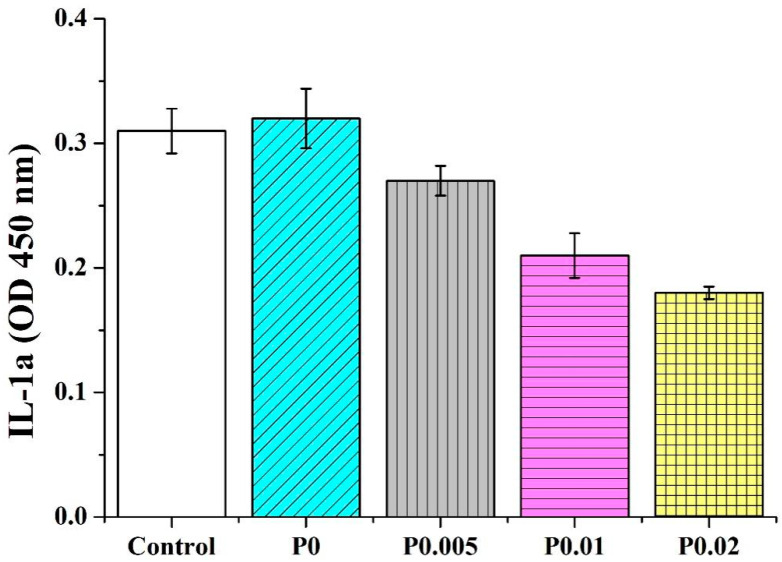
IL-1α inflammatory factor of nanofiber membranes as related to the *P. emblica* content.

**Figure 11:**
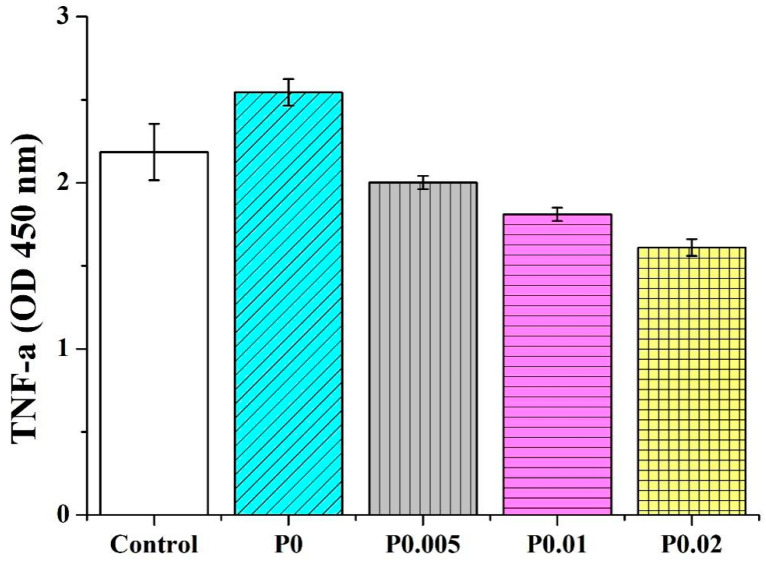
TNF-α inflammatory factor of nanofiber membranes as related to the *P. emblica* content.

PVP/CS/*P. emblica* nanofiber membranes are tested for their anti-inflammatory capacity. Two inflammatory factors (IL-1α and TNF-α) are used for the test. IL-1α and TNF-α are involved with inflammation and also trigger acute phase reaction proteins. A rise in the inflammatory factors inflicts cells with death, allergy, septicemia, or inflammatory reactions. As a result, the anti-inflammatory capacity tests with IL-1α and TNF-α as inflammatory factors can determine whether nanofiber membranes trigger the inflammation of cells and skins. Figure 10 shows that in the IL-1α test, the OD for the control group is 0.31 ± 0.01, while the ODs for PVP/CS, PVP/CS/*P. emblica* 0.005, PVP/CS/*P. emblica* 0.01, and PVP/CS/*P. emblica* 0.02 are 0.32 ± 0.02, 0.27 ± 0.01, 0.21 ± 0.01, and 0.18 ± 0.01. Figure 11 shows that in the TNF-1α test, the OD for the control group is 2.18 ± 0.17 while the ODs for PVP/CS, PVP/CS/*P. emblica* 0.005, PVP/CS/*P. emblica* 0.01, and PVP/CS/*P. emblica* 0.02 are separately 2.54 ± 0.08, 2.00 ± 0.04, 1.81 ± 0.04, and 1.61 ± 0.05. Based on the two anti-inflammatory capacity tests, the incorporation of PVP/CS induces the occurring inflammatory factors. In contrast, with an increase in the *P. emblica* content, PVP/CS/*P. emblica* groups show a declining trend in the inflammatory factor regardless of whether it is IL-1α or TNF-α. Specifically, the PVP/CS/*P. emblica* 0.02 demonstrates the optimal inhibition efficacy. It is proved that the nanofiber membranes do not cause an inflammatory reaction and that the incorporation of *P. emblica* can further reduce the presence of inflammatory factors [46].

## 4. Conclusions

In this study, electrospinning is employed and successfully creates PVP/CS/*P. emblica* nanofiber membranes to serve as dry facial masks with excellent melanin-confining effects and biocompatibility. The test results show that the maximal *P. emblica* extraction rate is 29.0% with an optimal extraction time of 12 h. However, the tyrosinase inhibition rate is adversely affected by a longer extraction time, which suggests that there are more different extracts or impurities. In addition, the resulting nanofiber membranes exhibit evenly distributed nanofibers and the PVP/CS/*P. emblica* 0.02 wt% group exhibits a diameter of 165.33 ± 51.66 nm. The nanofiber membranes also demonstrate good hydrophilicity with a water contact angle of 77.25 ± 2.21°, which facilitates the interaction between the membrane and water while improving the degradation level. Moreover, the release rate test indicates that the nanofiber membranes can reach a *P. emblica* release rate of 96.73 ± 0.35% in fifteen minutes and *P. emblica* can be totally released in twenty minutes. Furthermore, PVP/CS/*P. emblica* nanofiber membranes have tyrosinase inhibition rates as high as 99.53 ± 0.45%, thus suggesting an excellent restriction on melanin synthesis. PVP/CS/*P. emblica* nanofiber membranes demonstrate biocompatibility with a lowest cell activity of ≥82.60 ± 1.30%. Regarding the cytocompatibility of PVP/CS/*P. emblica* nanofiber membranes, it is proved that the cells demonstrate a good morphology and adhesion degree. As for the anti-inflammatory capacity, PVP/CS/*P. emblica* nanofiber membranes show inhibition capacity against IL-1α and TNF-α inflammatory factors that are separately reduced to 0.18 ± 0.01 and 1.61 ± 0.05. This result substantiates the view that ill inflammation is absent. Serving as dry facial masks, PVP/CS/*P. emblica* nanofiber membranes demonstrate excellent melanin-confining, hydrophilic, biocompatible, and inflammation-free features, and can be developed for and applied to the cosmetics field.

## Figures and Tables

**Table 1:** Specifications of solution A, B, C, and D for tyrosinase inhibition rate.

Solution	A	B	C	D
Tyrosine	100	100	100	100
Extract	-	-	80	80
Water/Ethanol (solvent)	80	100	-	20
Tyrosinase	20	-	20	-
Total	200	200	200	200

**Table 2:** Extraction time and extraction rate for *P. emblica*.

Time (h)	6	12	24	48
weight (g) before extraction	10	10	10	10
weight (g) after extraction	0.58	2.9	2.53	2.5
extraction rate (%)	5.8	29.0	25.3	25.0

**Table 3:** Specifications of PVP/WCS/*P. emblica* nanofiber membranes.

	Fiber Diameter (nm)	Viscosity (cP)	Conductivity (μS)
*P. emblica* 0	214.27 ± 74.51	351.8	657
*P. emblica* 0.005	107.95 ± 27.74	397.1	1084
*P. emblica* 0.01	90.32 ± 29.84	421.6	1336
*P. emblica* 0.02	165.33 ± 51.66	467.3	1609

## Data Availability

All data relevant to the study are included in the article.
